# Ethical Issues in Uncontrolled Donation After Circulatory Determination of Death: A Scoping Review to Reveal Areas of Broad Consensus, and Those for Future Research

**DOI:** 10.3389/ti.2025.13992

**Published:** 2025-02-06

**Authors:** Anastasia Georgiou, Weiyi Tan, Mihnea I. Ionescu, Isla L. Kuhn, Zoe Fritz

**Affiliations:** ^1^ School of Clinical Medicine, University of Cambridge, Cambridge, United Kingdom; ^2^ Miami Transplant Institute, Jackson Health System, Miami, FL, United States; ^3^ THIS (The Healthcare Improvement Studies) Institute, University of Cambridge, Cambridge, United Kingdom

**Keywords:** transplant, uncontrolled donation, cardiac arrest, ethical considerations, systematic literature review

## Abstract

Uncontrolled donation after circulatory determination of death (uDCD) protocols are established in several countries with good outcomes. We reviewed the literature between 1997 and 2024 to identify ethical issues. 33 papers were identified. Several areas of continued ethical debate were delineated: the role of advanced life support techniques; the ethical acceptability of aortic occlusion balloons; the nature and timing of consent to organ preserving techniques; whether best interests can/should extend beyond individual bodily integrity in this context. Further empirical research and ethical analyses are needed in these domains. Broad consensus was identified on several issues including: decisions about termination of resuscitation and entry into a uDCD protocol should be made by different teams; at least 20–30 min of cardio-pulmonary resuscitation is required; a hands-off period of 5–7 min is required alongside continuous monitoring; organ preserving techniques should be as minimally invasive as possible; families should be approached early to discuss organ donation by trained staff; public knowledge and engagement about uDCD is poor and must be improved; transparency and informed consent are essential for potential uDCD organ recipients. To maintain transparency and encourage positive public engagement we propose a name change from uDCD to Organ Donation after Sudden Irreversible Cardiac Arrest (ODASICA).

## Graphical Abstract

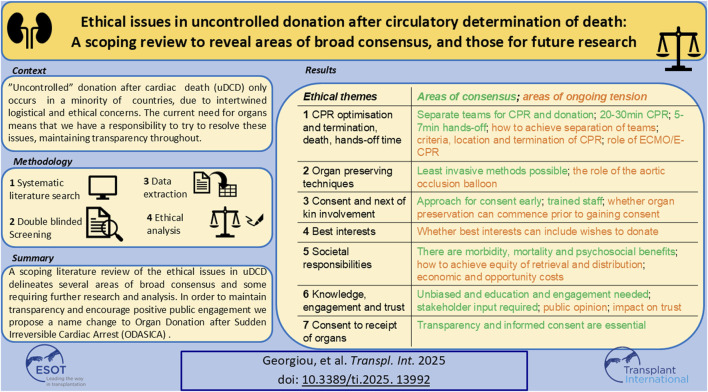

## Introduction

Organ donation has widespread public support and can provide great comfort to families after the death of their loved ones [1–3]. However, only a small number become organ donors [4]. In the UK for example, less than 0.5% of people who die under the age of 80 years become organ donors [4], a key reason for this being that there is no mechanism for people who die following out-of-hospital cardiac arrest to donate; only those who die in a “controlled” donation after circulatory death (cDCD) setting – on an intensive care unit, with planned withdrawal of treatment and immediate organ recovery – are eligible [5, 6].

So called “uncontrolled” donation after circulatory determination of death’ (uDCD) is possible in other countries after a witnessed cardiac arrest [7]. Cardio-pulmonary resuscitation (CPR) is started, and all efforts are made to revive the individual for at least 30 min; this may include mechanical CPR which allows safe transport to hospital with ongoing regular compressions. Once in the hospital, the treating team assesses whether there are any further interventions which might be successful; if there are not, then, as with all cardiac arrests in which return of spontaneous circulation has not been achieved, CPR is stopped, and the patient is pronounced dead. There follows a hands-off period before the transplant team starts efforts to preserve the organs [8].

The challenge with uDCD is to ensure that, after the death of the patient, donor organs are rapidly preserved before they are irreversibly damaged due to ischemic injury while still providing sensitive care to donor families. In France and Spain, normothermic regional perfusion (NRP), a minimally invasive technique to preserve the organs *in-situ* after death, is used routinely to facilitate uDCD donation and the resulting kidney transplant outcomes have been excellent [9, 10].

uDCD has been introduced around Europe with notable success in France [11] and Spain [12]: around 1000 successful kidney transplants have been performed since 2015 [7, 9, 13]. It is also practiced in Italy [14, 15] the Netherlands [16], Portugal [17], and Poland [18] and has been developed in Belgium [19], Russia [20], the US [21, 22], Taiwan [23] Korea [24], Austria [25] and the Czech Republic [26, 27] with different protocols [28] and with varying degrees of success. Systematic reviews have examined specific elements of the process and the associated outcomes including of Extracorporeal Membrane Oxygenation (ECMO) [29], preservation techniques [30, 31] and graft outcomes [32].

**FIGURE 1:**
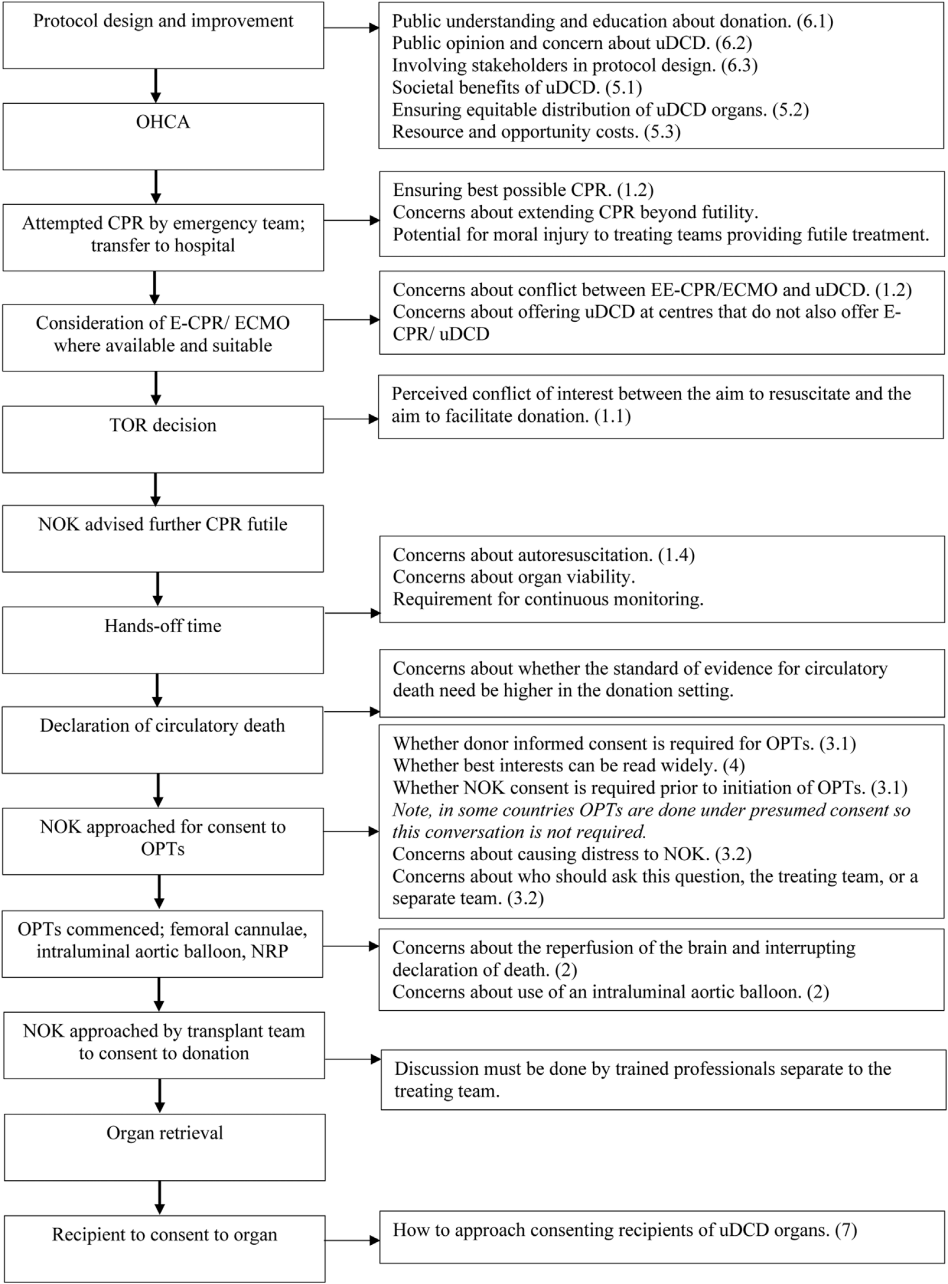
A visual representation of the ethical issues associated with uDCD. On the left, going from top to bottom, are the stages of a typical uDCD protocol in chronological order. On the right are the ethical issues associated with each stage. The numbers in brackets refer to the paragraph in which the issue is discussed. This figure was derived from our synthesis of the results in this paper.

Many overviews and editorials have been written on the challenges and ethical issues of both uDCD [33–39] and cDCD [40, 41]. Most ethical issues emerge from establishing the best way to act in the patient’s best interests when survival is no longer possible; there is perceived conflict between ensuring best end-of-life care and ensuring opportunity to donate organs. Figure 1 illustrates these points of conflict and ethical tension, alongside the uDCD process. See Table SA in the supplementary materials for a full Glossary and abbreviations associated with uDCD.

While many authors have identified ethical issues, there is insufficient empirical evidence or stakeholder engagement to develop a grounded understanding of normative claims, or what might be the “right” thing to do in several domains, particularly around conversations and consent. This contributes to a reluctance to initiate uDCD programs or even pilots, which itself creates an ethical issue given the shortage of organs for those who need them; around 5,000 people are waiting for a kidney transplant in the UK with an estimated 3 deaths per day are related to the shortage of donor organs [4], and over 100,000 are reported to be waiting in the USA [21, 42]. A uDCD program is predicted to allow the recovery of a significant number of organs per year; to not explore this route would be to deny these patients a life-saving donation and to deny others the opportunity to donate.

There appears, therefore, to be an ethical imperative to explore conducting uDCD; the International liaison Committee on Resuscitation recently conducted a thorough review of international protocols and concluded that *“All health systems should develop, implement, and evaluate protocols designed to optimize organ donation opportunities for patients who have an out-of-hospital cardiac arrest and failed attempts at resuscitation*” [43] but teams doing so must be fully informed its associated ethical issues so that they can address them within their protocols. Previous work has systematically delineated some specific issues: Bastami et al collated evidence surrounding healthcare providers’ and the public’s attitudes towards donation after cardiac death [44], Molina-Perez et al reviewed the role of families in deceased organ donation [45] and Schou et al reviewed ethical issues associated with extracorporeal life support [46] while Schiff et al have examined ethical issues associated with integrating ECMO and organ preservation in the USA [47]. However, there is no systematic review of original empirical studies or analyses on the ethical issues associated with uDCD.

We therefore undertook this review in order to (i) identify areas of ethical tension which need further research or stakeholder engagement and (ii) reveal areas of broad ethical consensus or empirical resolution. By doing this, we hope to be able to support those developing uDCD programs and direct researchers onto fertile ground.

## Methods

### Search

We systematically reviewed the literature for original, peer reviewed, articles on the ethical issues associated with uDCD.

**FIGURE 2:**
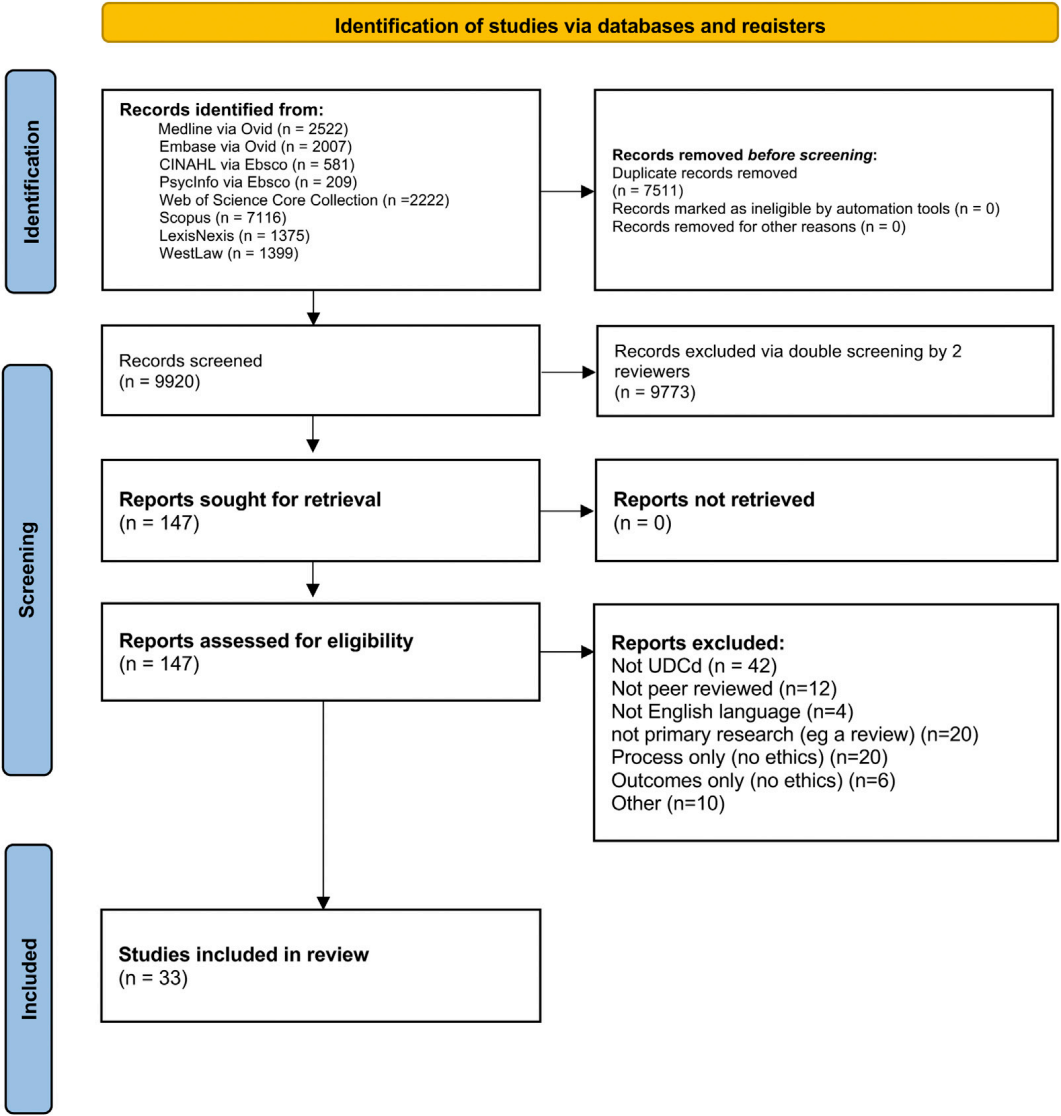
PRISMA diagram combining data from initial and rerun searches.

MEDLINE via Ovid, Embase via Ovid, CINAHL via Ebsco, Scopus, PsycInfo via Ebsco, LexisNexis, WestLaw and Web of Science Core Collection were searched using the following MeSH terms or other subject terms, and synonyms. The full search strategy can be found in the Supplementary Appendix, but can be summarized as: (1) out of hospital cardiac arrest terms OR uncontrolled AND (2) donation terms (adj5) AND (3) ethical OR legal terms The search was designed by a health librarian (IK) in collaboration with authors AG and ZF. Searches were run from 1st January 1997 until 1st April 2022 and re-run 30th May 2023 and again Sept 19th 2024. See Figure 2 for the PRISMA flow diagram.

### Screening

9,920 papers in total (7,605 in 2022, 1,035 in 2023 and 1,280 in 2024) were screened for inclusion/ exclusion, using the criteria shown in Table 1. Three authors, AG, MI and ZF, performed screening. Each paper was blindly screened by two of the three screening authors using Rayyan. The inclusion and exclusion criteria were applied to the title and abstract, and where the outcome was ambiguous, the full publication was read in full by all three members of the screening team and discussed until conflict was resolved.

**TABLE 1:** Inclusion and Exclusion criteria.

	Inclusion	Exclusion
Publication type	• Peer-reviewed • Journal, article, or chapter • Original analysis or data, e.g., original ethical analysis, empirical data pertaining to ethical issues about uDCD, or systematic ethical analysis • English language • Date from 01/01/1997 onward	• Not peer-reviewed • Opinion piece, commentary, review • No abstract • Not original analysis nor data (review article) • Systematic review (unless systematic ethical analysis)
Publication content	• uDCD • Ethics • Law • Policy analysis • Protocol analysis	• cDCD only • Pediatric (<18 years) organ donation • Non-human organ donation • Ethical issues relating to cellular level research • About process of donation only • About outcomes of uDCD only

We included two kinds of papers:

1. Papers which presented original peer reviewed ethical analysis (where ethical analysis is defined as: identifying questions regarding what is the “right” thing to do (or what we ought not to do); critically and reflectively examining [48, 49] different viewpoints; and presenting a reasoned argument, ideally with a normative conclusion of issues relating to uDCD) [50, 51].
2. Papers which presented empirical data pertaining to ethical issues about uDCD, in particular, studies examining perceptions of uDCD.

Systematic reviews were excluded from the review, although these papers were read as a further way of identifying relevant papers. Reviews, commentaries and opinion pieces were excluded. Papers which reported protocols, outcomes or processes of donation without ethical analysis were excluded. The nature of the included papers can be seen in Table 2.

**TABLE 2:** Publication characteristics of included papers.

	n/33	%
Country USA Canada UK Spain Switzerland Belgium Netherlands Italy Sweden Denmark Brazil	16 3 3 3 2 1 1 1 1 1 1	48 9 9 9 6 3 3 3 3 3 3
Region North America Europe (continent) Other	19 13 1	58 39 3
Publication type Systematic ethical analysis Primary ethical analysis Primary ethical and legal analysis Empirical analysis of public perception Empirical analysis of healthcare professional perception Empirical analysis of public and healthcare professional perception Empirical analysis of mass media campaigns Commentary on a protocol Primary ethical analysis and commentary on a protocol Case study, ethical and legal analysis	1 14 3 5 2 1 1 2 3 1	3 42 9 15 6 3 3 6 9 3
Date of publication 2001–2010 2011–2020 2021-May 2023	8 22 3	24 67 9

### Second Screening

All papers included on the basis of title and abstract were read in full by authors AG, MI and ZF. The inclusion/ exclusion criteria were applied blindly by each member. A full team meeting was held to agree final includes.

### Re-Runs of Search

The search was re-run in May 2023 using the same method as above, and again in October 2024 following peer review. A fourth member of the research team, author WT, read all papers included from both searches in full to ensure consistency of application of inclusion and exclusion criteria. Any conflicts were discussed and resolved as a team.

### Quality Assessment

In alignment with the PRISMA guidelines for scoping reviews, an assessment of whether quality assessment would be appropriate was undertaken [52]. Given the wide range and nature of articles identified, a formal quality assessment of included studies was not performed [53].

### Data Extraction and Analysis

The final included papers were read in full by authors AG and ZF and data was extracted.

A excel spreadsheet was created for extraction of data relating to both publication characteristics (title, date, author(s), country, article type, participants, limitations and content. Framework analysis was undertaken [54]: an initial coding framework was created based on themes identified in the background literature (see Supplementary Appendix for data extraction proforma).

Having familiarized themselves with the data in each paper, and iterated the initial coding themes further, authors AG and ZF independently coded each paper.

Further themes were added upon data extraction and discussion among the authors, who together charted, mapped (see Figure 1) and interpreted the data.

Some themes were grouped together for ease of understanding, with sub-themes being created. For example, the themes of “consent” and “‘relatives” were brought together under one heading, “consent and involvement of next of kin,” with two sub-themes, “informed consent to the use of organ preserving techniques” and “the approach to discussion of organ donation with next of kin” having emerged. In another example, “optimisation and termination of resuscitation” emerged as an important theme.

The final coding framework comprised seven broad ethical themes, along with subthemes. The frequency with which each ethical theme was raised in the literature was documented.

## Results

### Publication Characteristics

33 papers were included. Table 2 shows publication breakdown by country of origin, region, publication type and date.

### Ethical Issues

7 broad ethical themes, along with subthemes, were identified. Table 3 shows the proportion of papers addressing each issue. We explore each of these themes below and summarize the results in Table 4, highlighting areas of broad consensus and areas of ongoing ethical tension.

**TABLE 3:** Proportion of papers addressing each ethical issue.

Ethical issue				n/33	%
1	Optimization and termination of CPR, declaration of death and hands-off time	1.1	Conflicts of interest between treatment and transplant teams	15	45
		1.2	Optimization and termination of resuscitation	11	33
		1.3	Declaration of death	15	45
		1.4	Hands-off time	8	24
2	The use of organ preserving techniques			19	58
3	Consent and involvement of next of kin	3.1	Informed consent to the use of organ preserving techniques	17	52
		3.2	The approach to the discussion of organ donation with next of kin	22	67
4	Best interests, and whether this can extend beyond strict medical benefit			8	24
5	Societal responsibilities	5.1	Societal benefits of uDCD	25	76
		5.2	Distributive justice	6	18
		5.3	Resource cost	11	33
6	Public and professional knowledge, opinion, engagement and trust	6.1	Public understanding, education, and transparency	21	64
		6.2	Public opinion and concerns about the uDCD process	21	64
		6.3	Involvement and engagement	23	70
7	Informed consent to the receipt of organs from uDCD donors			2	6

**TABLE 4:** Summary of results.

Ethical issue				Areas of consensus	Areas requiring further research
1	Optimization and termination of CPR, declaration of death and hands-off time	*1.1*	*Conflicts of interest between treatment and transplant teams*	Decisions about TOR and uDCD entry should be made by different teams	Ensuring true separation between teams
		*1.2*	*Optimization and termination of resuscitation*	At least 20–30 min of CPR.	Factors precluding TOR. Location of TOR. Role of E-CPR/ ECMO.
		*1.3*	*Declaration of death*		Standard of evidence of circulatory death
		*1.4*	*Hands-off time*	At least 5–7 min alongside continuous monitoring	
2	The use of OPTs			The least invasive methods possible should be used	Ethical acceptability of aortic occlusion balloons
3	Consent	*3.1*	*Informed consent to the use of OPTs*		Whether consent to OPTs is covered under general consent to donation Whether OPTs can be commenced prior to family consent
		*3.2*	*The approach to the discussion of organ donation with next of kin*	Families can be approached early, with sensitivity and respect and by trained staff	
4	Best interests				Whether best interests can be read widely at the population level to include wishes to donate
5	Societal responsibilities	*5.1*	*Societal benefits of uDCD*	uDCD will increase the organ pool and has other psychosocial benefits	
		*5.2*	*Distributive justice*	uDCD organs must be recovered and distributed in an equitable way	Ensuring equity of recovery and distribution
		*5.3*	*Resource cost*		Short and long term financial and opportunity costs of uDCD.
6	Public and professional knowledge, opinion, engagement and trust	*6.1*	*Public understanding, education, and transparency*	Public knowledge about uDCD is poor and must be improved via unbiased education	
		*6.2*	*Public opinion and concerns about the uDCD process*		Public opinion on uDCD in different intersections of society Impact of uDCD on trust
		*6.3*	*Involvement and engagement*	Public education and stakeholder engagement is imperative Debate should be facilitated	
7	Informed consent to the receipt of organs from uDCD donors			Transparency and informed consent are essential	

### Optimization and Termination of CPR, Declaration of Death and Hands-Off Time

An overarching ethical challenge that was identified throughout the literature was the perceived tension between maximizing the chances of successful resuscitation for the patient who has arrested and maintaining organ viability if resuscitative attempts were to fail.

#### Conflicts of Interest Between Treatment and Transplant Teams

Many authors recognized a potential for conflicts of interest (or perceived conflicts of interest) at several stages of the uDCD protocol; if one team, or several closely linked teams, make(s) decisions about resuscitation, termination of resuscitation (TOR), declaration of death and recruitment into the uDCD protocol, questions about the quality of resuscitation, whether all efforts were made to save the patient’s life [8, 55] and about financial incentives [55, 56] are raised. Individual physicians may be placed in positions of conflict [55, 56] and perceived conflict of interest can erode trust in the donation system and medical system more broadly [57]. These concerns are reflected in quantitative data: Goudet et al found that a majority of healthcare respondents in their multicenter survey thought there is conflict of interest between saving lives and saving organs in the uDCD context [58].

Many proposed separating the roles [8, 21, 59, 60] although it was recognized this would not eliminate conflict if the teams are in contact [8, 21] and presents logistical and resource challenges [8, 21, 22, 59, 60].

#### Optimization and Termination of Resuscitation

It was universally accepted that CPR should not be terminated until it was clear that continuing would be futile for the patient. There is insufficient research to recommend a specific duration of resuscitation [61] and no internationally accepted guidance [58, 61–63] but most protocols mandate at least 20–30 min [8, 22].

Beyond traditional attempted CPR, several authors considered the role of Extracorporeal CPR (E-CPR) and Extracorporeal Membrane Oxygenation (ECMO) to ensure that optimal CPR had been delivered before TOR. Authors questioned whether doctors might be choosing between attempting E-CPR/ECMO (or directing a patient towards a center that provided this) and consideration of donation (or directing a patient towards a center that delivered this). ECPR is not yet widely available and evidence of its efficacy in the out-of-hospital cardiac arrest setting is still being gathered [55, 59, 61, 63]. Authors questioned whether the inclusion criteria for ECMO/E-CPR and uDCD are sufficiently similar for there to be a conflict [8, 55, 64], whether allowing uDCD without ECMO/E-CPR may disincentivize development of the latter [59, 60] and erode public trust [64] and whether insisting that all uDCD centers participate in ECMO/E-CPR practice or research will hinder donation, frustrate donor wishes [59] and create significant numbers of vegetative patients [65]. One of the included papers offered quantitative data; Goudet et al in their survey of 1057 hospital staff found that 20% of respondents thought that donation after circulatory death protocols should be suspended until precise indications for ECMO/E-CPR in refractory cardiac arrest have been defined [58].

There was consensus that TOR should be prohibited out of hospital while excluding reversible causes, i.e., if a shockable rhythm is present [8]. Ave et al went further, cautioning against TOR in non-shockable rhythms too as in the out-of-hospital cardiac arrest setting fine ventricular fibrillation and pulseless electrical activity could be missed; they considered the use of echocardiography at the hospital to rule this out [8]. Some protocols make TOR decisions upon arrival in the hospital [60], but some make them in the out of hospital setting, and then provide organ-preserving CPR during transit to the hospital [8, 66] whereupon death will be declared. In these latter cases there are concerns that the quality of CPR might be “subconsciously” compromised during transport [61].

#### Declaration of Death

In the circulatory determination of death, it is said that death is being declared at the permanent stage and that irreversibility will rapidly and inevitably ensue as no methods aiming at resuscitation will be performed [8, 59, 63, 65, 67–72]. Debate in terminology about declaration of death centers on these concepts of permanence (that circulation *will* not be restored) and irreversibility (that circulation *cannot* be restored); see Supplementary Table SB for further details. Some authors suggested that declaration of death in possible donation circumstances may require a higher standard of evidence of circulatory cessation than in non-donation circumstances because the consequences are greater for the patient [68]. For example, rather than relying on mechanical asystole it may be necessary to prove absence of circulation via arterial line, arterial doppler or echocardiogram [8]. The approach varies considerably in different countries [8, 71].

Conversely, some authors argued that declaration of death in the uDCD setting is less complex as by definition the patient will have undergone rigorous resuscitative efforts known to have failed [65, 66, 69]. Survey data is inconclusive, with some studies associating donation after circulatory death with greater perceived certainty of death than Donation after Brainstem Death (DBD) [71] and others finding the opposite [70].

A common theme in the papers was to comment on logical and semantic inconsistencies that have emerged over the years as new definitions of death were introduced to facilitate new donation practices [65, 71] (in one case referred to as “gerrymandering” [65]); death was historically defined by cardiac, or circulatory, criteria and the concept of brain death was introduced in the 1960s to allow DBD. See Supplementary Table SB for a summary of the changing definitions of death.

##### Hands-Off Time

Debate about the length of hands-off time – the period between termination of resuscitation, declaration of death, and initiation of insertion of cannulae for organ preservation – focused on a tension between concerns about case reports of autoresuscitation (also known as Lazarus phenomenon) [8, 59, 61, 68] and maximizing organ viability [59]; too short a period risks not giving opportunity for autoresuscitation and too long a period risks reduced organ viability.

Ave et al referenced Hornby’s systematic review of case reports of autoresuscitation: they are small in number, and most have occurred at under 5 min; those that occurred after that did so in the absence of continuous monitoring [8]. In view of this, Parent et al concluded that risks of autoresuscitation after 10 min are extremely low, and continuous monitoring would pick up those that occurred [59].

France and Spain have a hands-off period of 5 min [8]; Goudet et al, based on a survey of healthcare professionals, suggested a minimum of 2–5 min no touch time is necessary [58].

### The Use of Organ Preserving Techniques

Once death has been declared, and a hands-off period observed, organ preserving techniques (OPTs) are instigated. Broad ethical issues include risk of resuming brain circulation [8, 63, 65, 69, 73] and retroactively negating declaration of death [8, 68, 70], violation of bodily integrity [8, 58, 62, 72, 74], resource cost [8, 72] and stress for the treating physicians [8].

Violation of bodily integrity was the most cited with a common theme: there was consensus that the least invasive methods possible should be used to preserve the opportunity to donate [59, 62, 74–76]. Bruce et al’s study of emergency department (ED) patients and relatives found that a majority felt that insertion of groin tubes, CPR and ventilation were acceptable as OPTs, if there was as little invasion of the body as possible [75] and Goudet et al’s survey found that majority of respondents did not consider cannulation as a “bodily integrity alteration” [58]. Volk et al also found support for OPTs; 80% of their participants expressed support for a rapid organ recovery where they live [57].

Many authors commented on the ethical issues raised by specific techniques. Use of NRP (ECMO to perfuse the organs only) is defended by some [65, 72] as an essential means of preserving donation opportunity with good outcomes for kidneys [65] but specifically opposed by others [8, 63, 68, 69]. Use of an aortic occlusion balloon was described by some as a responsible method of facilitating organ preservation whilst preventing perfusion of the brain [60, 65, 67, 77] while others were concerned that it might render a physician complicit in a patient’s death [8, 63, 68, 70]. Use of cold preservation solution was opposed by two authors for reasons of poorer outcomes [65] and interference with determination of death [8] but supported by others as it carries less risk of brain reanimation [68]. Dubois et al in their survey of 70 members of the public found that 72% expressed support of a law permitting organ cooling in order to preserve organs [78].

### Consent and Involvement of Next of Kin

Given the time pressures of instigating OPTs, several authors explored the issues with consent, and attempted to determine the optimal timing, place, and content for conversations with relatives of the deceased which are both respectful of autonomy and compassionate. Volk et al found that hypothetical family consent to donation in uDCD settings was high [69% (95% CI 65%–73%)] when compared with cDCD [70% (95% CI 66%–75%)] and DBD [66% (95% CI 62–71)], however that participants were less confident in making donation decisions about a relative when compared with themselves (71% and 75% respectively) [57].

#### Informed Consent to the Use of OPTs

While some authors argued that consent for OPTs is covered under general consent for donation [58, 60, 62, 69, 79], most felt it was not: authors argued most frequently that the public are not well enough informed about what OPTs involve [8, 73, 80] and, to a lesser extent, that OPTs are not done strictly for the patient’s direct benefit [63, 80] and that people’s views on OPTs may be too nuanced to be summarized in one binary decision [81]. Several authors therefore proposed that specific consent was needed for OPTs given that they damage bodily integrity [8, 58] and could potentially violate patient autonomy [58].

Four papers provided survey data on the need for and optimal timing of family consent for OPTs (see Table 5) [57, 58, 75, 78]; a slim majority in three papers felt it was acceptable to proceed with OPT prior to family consent, but only 17% of participants in Volk’s 2010 study thought it was acceptable to proceed “in the absence of family consent or a known donor card” [57]; this study took place in the setting of an “opt in” system.

**TABLE 5:** Survey results on the acceptability of commencement of OPTs prior to gaining family consent.

	Commencement of OPTs while family consent is being sought IS acceptable	Commencement of OPTs while family consent is being sought is NOT acceptable	Unsure
Dubois et al [78]	49%	39%	12%
Goudet et al [58]	46.8%	42.5%	10/7%
Volk et al [57]	17% (95% CI 13%–20%)	Not available	Not available
Bruce et al [75]	48% (groin tube), 51% (CPR), 57% (ventilator)	28% (groin tube), 24% (CPR), 21% (ventilator)	24% (groin tube), 25% (CPR), 22% (ventilator)

In support of commencing OPTs prior to family consent were arguments that OPTs in uDCD are no more invasive than interventions done in the DBD setting [74], that it is the only way to preserve the family’s opportunity to make their own decision [79] and that to do otherwise renders protocols logistically impossible [21, 22, 67, 73]. Light et al and Wall et al reflected on their experiences in Washington, D.C. and New York respectively in which the requirement for family consent was the main barrier to success [21, 22, 67]. Other authors supported use of OPTs prior to family consent but only if the techniques are minimally invasive [59, 82]. Some argued strongly for the opposite: that OPTs should only be applied once both donor consent *and* family consent have been confirmed [57, 69]. Arguments for this approach included reducing mistrust [78], respect for autonomy [63, 83], reduction of resource cost [83] and reduction of family distress [60].

Two alternative systems were suggested: Moorlock et al proposed a detailed anticipatory consent form with which people can learn about and communicate their nuanced views on the complexities of OPTs [81] and Verheijde et al questioned whether a system of mandated informed decision making would be best [63]. These models for anticipatory specific consent would overcome concerns that specific consent for OPTs would render uDCD protocols logistically unworkable in an emergency setting [8, 21, 67, 76] thus reducing the number of available donors.

#### The Approach to the Discussion of Organ Donation With Next of Kin

Most papers found an acceptance for conversations about donation with next of kin to happen early in the acute setting. Wall et al described that, in the New York protocol, families were not offended by being asked soon after witnessing their loved one’s unexpected death [67]. Bruce et al, in their study of 200 members of the public, found that most people (54%) were willing to discuss donation soon after death in the ED [75], and that there is no difference in the number willing to discuss donation after circulatory death in ED (as is the case in uDCD) compared with brain death in ITU (72% in both cases, p = 0.146) [75]. Consistent with this, Wind et al found that consent rates were higher in patients who had an unexpected death than in those who had an expected death (61% and 45% respectively, P = 0.007) [82]. These empirical studies go some way to addressing Light’s concerns that it may be hard for families to cope with a sudden loss and the question of donation at the same time [22], although time constraints in contacting families may present a logistical barrier [22].

There was consensus that families should be approached with sensitivity and respect, by staff with specialist training [75, 80].

### Best Interests, and Whether This Can Extend Beyond Strict Medical Benefit

In uDCD protocols the patient will lack capacity and decisions must be made in their best interest [84, 85]. Best interests decisions must be made on a case-by-case basis [80, 81], but often little is known about the individual’s desires in the emergency situation: best interests decisions are therefore based in the first instance on population level knowledge.

If the concept of best interests is taken in its narrow, medical sense, uDCD becomes ethically challenging because OPTs are invasive and done beyond the point at which there will be medical benefit to the patient. Potential harms to the patient are physical [72, 73, 80], and non-physical e.g., treating the patient as a means to an end [73], violation of a deep desire to not donate [73], distress to the family [80], and impact on the dignity of a person [62]. Moorlock et al and De Lora et al questioned whether any harm can be inflicted on a nearly dead patient [80, 81] but concluded that there is a duty to treat cadavers with respect [80], and that respect for dead persons and posthumous wishes is an established ethical concept [81].

Several authors suggested that best interests should be interpreted more broadly than considering physical integrity [72]. Arguments included that best interests are now accepted as extending beyond the strictly clinical [80] and include fulfillment of wishes to donate [73, 80], promotion of dignity for example by “favoring the accomplishment of their life project” to donate [62] and permitting altruism in end of life planning [73]. OPT may preserve the family’s opportunity to make their own informed decision about donation [80] and preserve the autonomy of those patients who turn out to have expressed wishes to donate [72]. Without OPTs, the opportunity to donate is lost [72, 74, 78].

### Societal Responsibilities

Several papers considered the ethical duty to consider responsibilities to society as well as to the individual.

#### Societal Benefits of uDCD

The most frequently raised benefit was the increased number of organs [21, 22, 55, 57–60, 63, 65–69, 71, 72, 74–79, 82, 83] and therefore reduced morbidity and mortality, which is widely seen as a “societal good” [74] with only one of the included papers disagreeing [63]. Several papers give data on the organ pool, providing international evidence of the potential benefit of introducing a uDCD program [57, 63, 65, 67, 69, 78].

Other societal benefits may include psychosocial benefits [73], comfort to grieving families [73], reduction in coercive or illegal organ practices if more legitimate organs are available [66], and economic benefits through taking patients off costly dialysis [72, 73] and returning them to economic activity [73].

#### Distributive Justice

Several papers addressed concerns that organs may be recovered and distributed in an inequitable way on the uDCD pathway. It was noted that uDCD is likely to be disproportionately available in large inner-city hospitals [64] and that these usually serve socioeconomically disadvantaged populations [55, 61, 64] in which cardiac arrest [61] and violent or traumatic injury [22, 55, 61, 64] is more common. Authors commented on the disproportionate representation of ethnic minorities in donor populations [55, 64], resulting in complaints that the system is biased [55], and on minority group members expressing significant mistrust and suspicion toward organ donation [64]. Moorlock et al noted that uptake of advance care planning has been found to be lower among older people from ethnic minorities [81] and questioned whether their own proposal to introduce a comprehensive consent form would exacerbate existing inequalities in organ donation.

Ave et al argued that socioeconomically advantaged patients who have better access to health resources may be more likely to receive a uDCD transplant [61], and a case of perceived unjust allocation of organs to a wealthy, prominent figure was noted [64]. In contrast, Wall et al discussed data showing that underserved communities are disproportionately affected by conditions leading to renal failure and therefore receive more organs [66]. Allocation by age of recipient was raised by Light et al; they noted that recent moves to use expanded criteria donors have not benefitted younger, healthier recipients, only older ones, but that uDCD programs may.

#### Resource Cost

uDCD protocols are highly resource intensive [8, 22, 60, 66, 67, 69, 72, 73, 83, 86] in view of the equipment, personnel [60], transport [8] and training costs. The opportunity costs (for example in ambulances being unavailable for other sick patients because they are being used to transport patients into ED who might otherwise have been declared dead out of hospital [69]) associated with a uDCD program are significant although not universal and would vary depending on individual center capacities [73]. Some papers discussed ideas for mitigating opportunity costs, for example having separate ambulances for potential donors [83] or limiting uDCD to in-hospital settings only [69]. Several authors postulated that the overall uDCD costs would be mitigated in the long term with fewer patients on dialysis [69, 73] and with the development of economies of scale as projects expand [66].

### Public and Professional Knowledge, Opinion, Engagement and Trust

Public and clinician trust in organ donation and in the wider medical system is imperative and links to our societal responsibilities [64]. If people do not trust the system they are less likely to donate and support transplantation as a whole [69].

#### Public Understanding, Education and Transparency

Several authors reported that current public understanding of uDCD is poor [8, 58, 86] and that uDCD protocols can differ substantially from common ideas of what donation involves [8]. In France and Spain no program of public information was conducted; the opt-out system was introduced without data on public opinion [8]. Bednecko et al found that 60% of their participants in Brazil didn’t know about donation legislation [86], and Goudet et al found that a majority of their survey participants in France considered the paucity of public information to be unacceptable and possibly reflective of concerns the medical community itself has about uDCD [59].

Most authors agreed that more substantial public information is needed [58]. Understanding allows people to make informed autonomous decisions [8, 80], helps to avoid mistrust in the system [8, 64], ensures that policies are ethically acceptable [80], improves enrollment rates [66] and may even reduce illegal organ trade [66]. Education must be transparent and accurate [8, 56, 69, 72], comprehensive [59], be directed toward the local community [64], include information on how to opt-out [8, 80, 81], and be deliverable through diverse media [22].

Rady et al emphasized the difference between education (providing information) and propaganda (communicating with a view to influence) and suggested separation of the governmental agency responsible for organ transplantation practice from the agency responsible for organ donation campaigns [56]. They based this proposal on concerns about bias, inaccuracy, misinformation, and undeclared conflicts of interests perceived in other campaigns [56] and a noted discrepancy between controversies happening in the scientific communities, and public messages which suggest no such controversies exist [70]. Moorlock et al’s proposal involves integrating education materials and specific elements of consent [81].

#### Public Opinion and Concerns About the uDCD Process

Several papers provided empirical data on opinions toward uDCD, with four finding equal support [57, 58, 71, 75], and two finding less support for uDCD than other types of donation [70, 86].

uDCD protocols have potential to engender mistrust due to the use of OPTs without consent [78], concerns that the patient may not actually be dead [56, 70, 72] and that there is violation of the prohibition against interfering with a dead body [66, 69, 83]. Several authors raised that mistrust is disproportionately felt by ethnic minorities [55, 64, 78]. Perceived conflict of interest between treatment and donation can cause mistrust toward, and between, healthcare professionals [63, 64] owing to the perception that organ donors may receive less aggressive life-saving care [59, 73], although Volk et al’s study reported that “the idea of a rapid organ recovery program did not significantly increase fears that signing an organ donor card would make doctors not try as hard to save their life” [57].

#### Involvement and Engagement

Most authors agreed that, in order to achieve transparency, accountability [64], and sustainable program success [67] numerous stakeholders must be consulted, including the government, the transplant network, public health, medical and ethical communities, the public [60, 65], secular and religious community organizations [59, 67, 71], community boards representing multi-ethnic populations [66], emergency practitioners [49] and specific local stakeholders [73]. Several authors also suggested that open and clear debate among both healthcare professionals and the public should be facilitated [62, 83], and Dubois et al raised the importance of monitoring uDCD protocols, suggesting that review boards be set up to assess adherence to policy and ensure accountability [78].

Delora et al explored how the mandate for uDCD protocols is established. They delineate the difference between allowing the initiation of OPTs on the basis of presumed consent via parliament legislation, as was the case in the Netherlands [80], and via governmental decree, as was the case in Spain [80]. The former involves democratic, accountable debate whereas the latter does not [80].

### Informed Consent to the Receipt of Organs From uDCD Donors

Finally, two of the included papers raised the issue of how recipients of organs procured via uDCD should be consented for a transplant [8, 76] and how much information they should be given on the source of the organ. Data suggests that the long-term outcomes of organs transplanted in uDCD protocols are as good as those transplanted in DBD protocols [62] and in cDCD protocols [22, 59] however there is evidence to suggest that there is a higher rate of shorter term complication, i.e., delayed graft function in uDCD [8]. Transparency and informed consent are essential, particularly in areas where uDCD is in development.

## Discussion

We have reviewed the literature on uDCD and identified areas of broad consensus and areas of ongoing ethical tension. Teams proceeding in piloting uDCD protocols, should do so with concurrent outcome and ethical evaluation. Several issues require further analysis; we will focus on four with reference to wider empirical, philosophical, and ethical literature.

### Optimizing Outcomes for the Individual and for the Organs

In attempting CPR, the primary intention is to regain spontaneous circulation and neurological recovery for the patient [87]; a secondary effect is optimal perfusion of the organs for transplantation should CPR be stopped. In transferring a patient to hospital, the primary intention is to ensure a comprehensive assessment of the irreversibility of the condition; a secondary effect is ensuring efficient organ recovery should CPR be stopped. Therefore, by optimizing CPR and transferring an arrest patient to hospital, the treating team is both optimizing patient outcomes and organ viability; they are not choosing one over the other [88].

Further, once CPR is stopped, 5 min of hands-off time followed by 5 min of continuous monitoring while cannulating results in a total of 10 min before the aortic occlusion balloon is inflated and NRP started. While case studies of autoresuscitation have been reported after termination after CPR, most are associated with confounders and a recent systematic reviews showed that it is extremely rare for them to take place after 10 min; none have occurred in the situation being proposed, with continuous monitoring for 5 min [89–91].

Some caveats do remain. First, we found consensus that decisions about TOR and entry into a uDCD protocol should be made by different teams; research into the logistics and outcomes of this is needed. Second, we found disagreement over whether the level of resuscitation should extend beyond advanced life support to ECMO/E-CPR; more research is needed into the benefits of these techniques in the out-of-hospital cardiac arrest setting and the impact that having uDCD without ECMO/ E-CPR may have on outcomes and on community trust.

### Best Interests Can Extend Beyond an Individual’s Lifetime

Authors of the included papers disagreed whether best interests in the uDCD context extend beyond the strictly clinical and beyond the individual’s lifetime. UK law supports a broad reading of best interests, and in the cDCD debate it has been argued that “where a patient would wish to donate, measures [that] are necessary for organ donation to proceed … serve, rather than deny, the best interests of a patient” [92] and are therefore autonomy respecting. The difficulty is that within an opt out system (and without Moorlock and Draper’s ambitious proposal of mandated anticipatory consent) [81], the specific wishes of most individuals are not known.

Although best interest decisions are, by definition, person specific, they are often initially made on population level knowledge. For example, a person found in cardiac arrest will be subject to CPR while further information on their wishes is sought [93]. This logic can reasonably be applied in the donation setting given that a majority of the population – with the information currently available to them – would like to donate [2]; while information is being sought about a person’s wishes, it may be in their best interests to cannulate and start NRP to preserve opportunity for donation.

### The Role of the Aortic Occlusion Balloon

There has been significant discussion about the role of the aortic occlusion balloon in All forms of DCD [8, 60, 63, 65, 67, 68, 70, 77, 94, 95]; this discussion is intimately associated with the definition of death [47, 96], and the philosophical debate around the ethical relationship between acts and omissions [97].

The device is required because a secondary effect of starting NRP is to resupply blood and oxygen to the brain to the same level of the attempted CPR. There is no evidence that this level of recirculation is likely to facilitate awareness or pain, but it is impossible to say for certain that there is no perception. Therefore, to avoid an unintended harm, the aortic occlusion balloon is inserted to prevent all circulation to the brain and maintain a peaceful death.

A recent prospective study by Royo-Villanova et al showed that when the thoracic aorta was blocked with an aortic occlusion balloon the mean intracranial arterial blood pressure at the circle of Willis was the same during circulatory arrest as it was following NRP being started, confirming that this technique works to stop brain perfusion [98]; this study should provide reassurances to those to those who were concerned about the efficacy of the aortic occlusion balloon.

Some have expressed concern that insertion of an aortic occlusion balloon in order to block circulation to the brain is itself an act which hastens death [8, 59, 63, 68]. In the cases we are considering, however, the patient has already died; their heart has stopped, there has been no responsiveness with CPR, and a multidisciplinary team has recognized the futility of further efforts. We agree with Schiff et al who say: “this is similar to *ex vivo* perfusion, in which perfusion is restored to the recovered organ to increase transplant viability, while the process towards loss of brain function in the donor body is allowed to continue.” [47] On this view, the aortic occlusion balloon is acting to minimize harm, while maximizing the individuals’ potential to donate.

### Transparency and Public Engagement

The above conclusions - namely that the interests of both resuscitation and donation can be simultaneously respected; that best interests apply posthumously and can be read broadly; and that an aortic occlusion balloon is in a patient’s best interests – are contingent on transparency and public engagement [8, 47, 56, 58, 59, 63, 64, 69, 72, 80, 83, 99]. If population level data is to be used to inform initial presumptions about what is in a patient’s best interests, public attitudes must be regularly surveyed and assumptions cannot be made [8, 56, 62, 80, 83].

There is some nervousness surrounding public discussion of the details of uDCD. While public attitudes toward donation are predominantly positive, there is an awareness that one bad media story can change views and potentially cost lives if it results in people opting out [21, 22]. The risks are increased when - as needs to be done - relatives are being asked to consent not only for transplant but for research into a new way of undertaking transplant. This nervousness is justified given the stakes, but it is a reason for ensuring that information about transplantation is understandable and widely available; hiding information is much more likely to erode trust in the long term. We should borrow from the World Health Organization’s advice on transparency in public health emergencies: information must be *“factually accurate, easily understood by the intended audience and presented in a manner that promotes adoption of the desired behaviors”* and we *must “promote trust by being forthcoming and open* …, *including the evidence and assumptions used by authorities in making decisions, the manner in which those decisions are being made and by whom.”* [100]

Finally, if public engagement and trust are to be sought, an alternative name to “Uncontrolled Donation after Cardiac Death” should be considered. The name derives from differentiating it from the controlled setting of an intensive care unit with planned withdrawal of treatment, but to those who don’t know this history, the term “uncontrolled” implies chaos and lack of regulation. As O’Rourke et al state, “who would wish to be involved in an “uncontrolled process”?” [101] A name that clearly describes the practice could be considered: Organ Donation After Sudden Irreversible Cardiac Arrest (ODASICA), or some other clearly descriptive explanation, may go some way towards engaging the public.

## Strengths and Limitations

We conducted a scoping review of the literature: the selection of papers was systematic, and blinded. Data was extracted on a standardized template and more than two authors read each paper to ensure agreement on the relevant themes.

Our study has limitations. There is some subjectivity in determining the difference between a review article and one which provides “original ethical analysis” of uDCD; we chose not to include review, opinion or comment articles as many of these were summarizing the articles which were already included. We may have missed some potentially relevant literature that did not fit the search terms, although this was minimized by snowballing the references which were identified. The review is based on published research literature and excluded operational or programmatic reports and book chapters which may have added valuable insights. The heterogeneous nature of the papers identified meant that it was not possible for us to evaluate quality of publications or provide many quantitative findings. The papers identified, however, provided rich material for a comprehensive review of the ethical issues associated with uDCD.

## Conclusion

uDCD – or Organ Donation after Sudden Irreversible Cardiac Arrest (ODASICA) – is a complex process which is unfamiliar to many; carefully considering the ethical issues involved at each stage is therefore critical. This review provides evidence of broad ethical consensus in many areas. Future protocols should acknowledge remaining areas of potential conflict and prospectively collect empirical evidence from relatives and clinicians to ensure greater understanding and transparency.

## Data Availability

The original contributions presented in the study are included in the article/Supplementary Material, further inquiries can be directed to the corresponding author.
