# Supplementary material for: Ethical Issues in Uncontrolled Donation After Circulatory Determination of Death: A Scoping Review to Reveal Areas of Broad Consensus, and Those for Future Research
Source: Transpl Int. 2025 Feb 6;38:13992. doi: 10.3389/ti.2025.13992 (PMC11840875; doi:10.3389/ti.2025.13992)
Supplement: Supplementary file 1 [file DataSheet1.pdf]

APPENDIX

Table SA: Glossary and abbreviations 1
Table SB: Summary of changing definitions of death 3
Search Strategy 3

Table SA: Glossary and abbreviations

Term	Meaning
**ALS**	Advanced life support: resuscitation techniques and protocols used by trained medical professionals to provide urgent treatment to patients in cardiac emergencies.
**Aortic occlusion balloon**	A technique used in NRP to ensure that perfusion of the organs is limited to those to be transplanted excludes of the brain. An intraluminal occlusive balloon is introduced into the aorta via peripheral cannulation and inflated after declaration of death and before the commencement of NRP.
**Autoresuscitation**	The return of spontaneous circulation after CPR has been attempted, deemed futile and terminated. It is also known as the Lazarus effect.
**Best interests**	When a person lacks capacity, decisions must be made in their best interests as per the MCA 2005 and the GMC's Good Medical Practice guidance.
**cDCD**	Controlled donation after circulatory determination of death: recovery of organ(s) after death has been declared by cardio-respiratory criteria criteria following the planned withdrawal of life support, usually in the intensive care unit setting (also referred to as Maastricht category 3).
**Cold preservation solution**	A technique used to preserve organs for transplantation; a cold preservation solution is infused into the organ and then stored at a hypothermic temperature.
**CPR**	Cardio-pulmonary resuscitation.
**DBD**	Donation after brainstem death: recovery of organ(s) for the purposes of transplantation after death has been declared by neurological criteria.
**DCD**	Donation after circulatory determination of death: recovery of organ(s) for the purposes of transplantation after death has been declared by cardio-respiratory criteria.
**Declaration of death**	Legal declaration of death performed by a doctor on the basis of either circulatory or neurological criteria.
**ECMO**	Extracorporeal membrane oxygenation: a technique used to provide cardiac and respiratory support to patients by removing deoxygenated blood from the venous system, externally removing carbon dioxide, oxygenating, and returning it to the arterial system.
**E-CPR**	Extracorporeal cardio-pulmonary resuscitation: use of ECMO in the context of cardiac arrest.

**Expanded criteria donor**	Donors of organs for the purposes of transplantation who are over the age of 60, or over the age of 50 with two of the following: hypertension, creatinine le, or death resulting from a stroke.
**GMC**	General Medical Council: independent regulatory body with duties set out by the Medical Act 1983.
**Hands-off time**	Also referred to as ‘no-touch time’, the time interval between termination of resuscitation and declaration of death. A minimum period is usually mandated in the organ donation context in order to exclude the possibility of autoresuscitation.
**MCA**	Mental Capacity Act 2005: legislation on capacity assessments and best interests decision making.
**NRP**	Normothermic regional perfusion: in-situ preservation of organs with oxygenated blood at normothermia using extracorporeal membrane oxygenation devices. Sub-types include abdominal NRP (A-NRP) and thoracoabdominal NRP (TA-NRP).
**Organ-Preserving cardio pulmonary resuscitation**	OP-CPR: CPR performed after the point at which further CPR is deemed to be futile. The purpose of OP-CPR is to maintain blood flow to the organs and thus preserve them for the purposes of transplantation.
**OPT**	Organ preserving techniques: practices implemented between declaration of death and organ recovery, aimed at preserving the quality of organs and minimizing ischemic injury. Examples of OPT are cold preservation solution and NRP.
**PEA**	Pulseless electrical activity: a non-shockable cardiac rhythm.
**Return of spontaneous circulation**	Return of spontaneous circulation: the resumption of cardiac and respiratory effort in a patient that has sustained cardiac arrest.
**TOR**	Termination of resuscitation: the decision to stop resuscitative efforts for reasons of futility.
**uDCD**	Uncontrolled donation after circulatory determination of death: recovery of organ(s) for the purposes of transplantation after death has been declared by cardio-respiratory criteria on arrival at the emergency department (also referred to as Maastricht category 1) or after unsuccessful resuscitation attempts (also referred to as Maastricht category 2).
**VF**	Ventricular fibrillation: a shockable cardiac rhythm.

Table SB: Summary of changing definitions of death

**Pre-1960s:**	**Death was diagnosed according to 'cardiac and respiratory' criteria(48) and organs typically came from non-heart-beating donors(54).**
**1968-1991:**	Organ donation expanded to include DBD, which required the development of 'brain' death as an alternative criterion(54), enshrined in the Uniform Determination of Death Act (USA)(48). Most organs were procured from DBD.
**1990s:**	There were new efforts to procure more organs, and cDCD was re-explored. Death was in these cases defined according to 'cardiac' criteria(48).
**2006**	There was a shift from cardiac to 'circulatory' determination of death, as per the Institute of Medicine in their 2006 report(48). The terms 'permanence' (circulation *will not* be restored) and 'irreversibility' (circulation *cannot* be restored) were introduced and the 'dead donor rule' (that the recovery of organs cannot precede or promote the onset of death(55) was considered respected because cDCD protocols preclude further resuscitation and the absence of cardiac activity is therefore permanent and will soon be irreversible(48).
**Current**	In the UK there is no statutory definition of death. Instead, the definition is based on common law acceptance of professional guidance. Professional guidance provides that death can be declared following either the (i) irreversible cessation of brainstem function or (ii) the irreversible cessation of cardiorespiratory function(83).

Search Strategy

Medline via Ovid: Ovid MEDLINE(R) and Epub Ahead of Print, In-Process, In-Data-Review & Other Non-Indexed Citations, Daily and Versions <1946 to March 31, 2022>

1	(UDCD* or DCD*).ti,ab.
2	((cardi* arrest or cardi* death or circulatory arrest or circulatory death or cardiopulmonary death or (cess* adj2 cardiopulmonary) or dead on arrival or maastricht or OHCA or (uncontrolled not uncontrolled trial*)) adj5 (donat* or donor* or harvest* or organ transplant* or procur* or recei* or recover* or rescu* or retriev* or tissue procurement or transplant*)).ti,ab.
3	(exp Heart Arrest/ or Cardiopulmonary Resuscitation/ or Out-of-Hospital Cardiac Arrest/) and (exp Transplants/ or exp "Tissue and Organ Procurement"/ or Transplants/)
4	1 or 2 or 3
5	(organ* or kidney* or liver* or heart* or lung* or multivisceral or graft or bloc or bowel or small intestine* or pancreas).ti,ab,hw,kf,kw.
6	(act or best interest* or beneficiar* or information shar* or challeng* or barrier* or facilitat* or practical* or code* or constitution* or difficult* or ethic* or fair* or ideal* or illegal* or immoral* or issue* or law* or legal* or legislat* or jurisprudence or litigat* or moral* or principl* or philosoph* or precedent or problem* or (public adj3 (opinion* or perception* or perceiv* or attitude*)) or regulat* or right* or standard* or statut* or unconscion* or unconstitutional or unethic* or unfair* or unlawful or value* or virtu*).ti,ab. or Ethics, Research/ or Ethics/ or Ethics Committees, Research/ or "Codes of Ethics"/ or Ethics, Clinical/ or Ethics, Medical/ or Jurisprudence/ or Morals/ or exp public opinion/ or exp legal aspect/

7	(exp Animals/ or (rat or rats or mice or mouse or pigs or pig).ti,ab.) not (humans/ and exp animals/)
8	4 and 5 and 6
9	8 not 7
10	limit 9 to yr="1997 -Current"

Embase via Ovid: Embase <1974 to 2022 March 31>

1	((cardi* arrest or cardi* death or circulatory arrest or circulatory death or cardiopulmonary death or (cess* adj2 cardiopulmonary) or dead on arrival or maastricht or OHCA or (uncontrolled not uncontrolled trial*)) adj5 (donat* or donor* or harvest* or organ transplant* or procur* or recei* or recover* or rescu* or retriev* or tissue procurement or transplant*)).ti,ab.
2	(exp *Heart Arrest/ or *Resuscitation/ or exp *"out of hospital cardiac arrest"/) and (exp *Transplantation/ or exp *organ transplantation/)
3	(UDCD* or DCD*).ti,ab.
4	1 or 2 or 3
5	(organ* or kidney* or liver* or heart* or lung* or multivisceral or graft or bloc or bowel or small intestine* or pancreas).ti,ab.
6	(act or best interest* or beneficiar* or information shar* or challeng* or barrier* or facilitat* or practical* or code* or constitution* or difficult* or ethic* or fair* or ideal* or illegal* or immoral* or issue* or law* or legal* or legislat* or jurisprudence or litigat* or moral* or principl* or philosoph* or precedent or problem* or (public adj3 (opinion* or perception* or perceiv* or attitude*)) or regulat* or right* or standard* or statut* or unconscion* or unconstitutional or unethic* or unfair* or unlawful or value* or virtue* or virtuous).ti,ab. or *research ethics/ or *Ethics/ or *professional standards/ or *medical ethics/ or exp *Jurisprudence/ or exp *Morality/ or exp *public opinion/ or *applied ethics/ or exp *law/
7	(exp Animal/ or (rat or rats or mice or mouse or pigs or pig).ti,ab.) not (human/ and exp animal/)
8	4 and 5 and 6
9	8 not 7
10	limit 9 to yr="1997 -Current"
11	limit 10 to embase

PsycINFO via EbscoHost

S9	s7 not s8
S8	DE "Animals" OR DE "Animal Limb" OR DE "Animal Offspring" OR DE "Female Animals" OR DE "Infants (Animal)" OR DE "Invertebrates" OR DE "Male Animals" OR DE "Pets" OR DE "Service Animals" OR DE "Species Differences" OR DE "Vertebrates" or TI(rat or rats or mice or mouse or pigs or pig) or AB(rat or rats or mice or mouse or pigs or pig)
S7	S4 AND S5 AND S6

S6	TI(act or "best interest*" or beneficiar* or "information shar*" or challeng* or barrier* or facilitat* or practical* or code* or constitution* or difficult* or ethic* or fair* or ideal* or illegal* or immoral* or issue* or law* or legal* or legislat* or jurisprudence or litigat* or moral* or principl* or philosoph* or precedent or problem* or (public N3 (opinion* or perception* or perceiv* or attitude*)) or regulat* or right* or standard* or statut* or unconscion* or unconstitutional or unethic* or unfair* or unlawful or value* or virtu*) OR AB(act or "best interest*" or beneficiar* or "information shar*" or challeng* or barrier* or facilitat* or practical* or code* or constitution* or difficult* or ethic* or fair* or ideal* or illegal* or immoral* or issue* or law* or legal* or legislat* or jurisprudence or litigat* or moral* or principl* or philosoph* or precedent or problem* or (public N3 (opinion* or perception* or perceiv* or attitude*)) or regulat* or right* or standard* or statut* or unconscion* or unconstitutional or unethic* or unfair* or unlawful or value* or virtu*) OR (((((DE "Ethics" OR DE "Bioethics" OR DE "Experimental Ethics" OR DE "Professional Ethics") OR (DE "Morality")) OR (DE "Best Practices")) OR (DE "Public Opinion")) OR (DE "Law (Government)" OR DE "Civil Law" OR DE "Criminal Law" OR DE "Laws")) OR (DE "Litigation")
S5	TI(organ* or kidney* or liver* or heart* or lung* or multivisceral or graft or bloc or bowel or small intestine* or pancreas) or AB(organ* or kidney* or liver* or heart* or lung* or multivisceral or graft or bloc or bowel or small intestine* or pancreas)
S4	S1 OR S2 OR S3
S3	TI(UDCD* or DCD*) OR AB(UDCD* or DCD*)
S2	(DE "Organ Transplantation" OR DE "Tissue Donation")AND (DE "Heart Disorders" OR DE "Angina Pectoris" OR DE "Arrhythmias (Heart)" OR DE "Coronary Thromboses" OR DE "Myocardial Infarctions")
S1	TI((cardi* arrest or cardi* death or circulatory arrest or circulatory death or cardiopulmonary death or (cess* n2 cardiopulmonary) or dead on arrival or maastricht or OHCA or (uncontrolled not “uncontrolled trial*”)) n5 (donat* or donor* or harvest* or "organ transplant*" or procur* or recei* or recover* or rescu* or retriev* or "tissue procurement" or transplant*)) or AB (((cardi* arrest or cardi* death or circulatory arrest or circulatory death or cardiopulmonary death or (cess* n2 cardiopulmonary) or dead on arrival or maastricht or OHCA or (uncontrolled not “uncontrolled trial*”)) n5 (donat* or donor* or harvest* or "organ transplant*" or procur* or recei* or recover* or rescu* or retriev* or "tissue procurement" or transplant*)))

CINAHL via EbscoHost

S9	S8 NOT S7
S8	S4 AND S5 AND S6
S7	(MH "Animals+") or TI(rat or rats or mice or mouse or pigs or pig) or AB(rat or rats or mice or mouse or pigs or pig)

S 6	TI(organ* or kidney* or liver* or heart* or lung* or multivisceral or graft or bloc or bowel or small intestine* or pancreas) or AB(organ* or kidney* or liver* or heart* or lung* or multivisceral or graft or bloc or bowel or small intestine* or pancreas)
S 5	TI(“act” or "best interest*" or “beneficiar*” or "information shar*" or “challeng*” or ”barrier*” or “facilitate”* or “practical*” or “code*” or “constitution*” or “difficult*” or “ethic*” or “fair*” or “ideal*” or” illegal*” or “immoral*” or “issue*” or “law*” or “legal*” or “legislat*” or “jurisprudence” or “litigat*” or “moral*” or “principl*” or “philosoph*” or “precedent” or “problem*” or (“public” N3 (“opinion*” or “perception*” or “perceiv*” or “attitude*”)) or “regulat*” or “right*” or “standard*” or “statut*” or “unconscion*” or “unconstitutional” or “unethic*” or “unfair*” or “unlawful” or “value*” or “virtue*” or “virtuous”) OR AB(“act” or "best interest*" or “beneficiar*” or "information shar*" or “challeng*” or ”barrier*” or “facilitate”* or “practical*” or “code*” or “constitution*” or “difficult*” or “ethic*” or “fair*” or “ideal*” or” illegal*” or “immoral*” or “issue*” or “law*” or “legal*” or “legislat*” or “jurisprudence” or “litigat*” or “moral*” or “principl*” or “philosoph*” or “precedent” or “problem*” or (“public” N3 (“opinion*” or “perception*” or “perceiv*” or “attitude*”)) or “regulat*” or “right*” or “standard*” or “statut*” or “unconscion*” or “unconstitutional” or “unethic*” or “unfair*” or “unlawful” or “value*” or “virtue*” or “virtuous”)
S 4	S1 OR S2 OR S3
S 3	TI(UDCD* or DCD*)OR AB(UDCD* or DCD*)
S 2	((MH "Heart Arrest+") OR (MH "Resuscitation, Cardiopulmonary+")) AND ((MH "Transplant Donors+") OR (MH "Pretransplantation Period") OR (MH "Organ Procurement+") OR (MH "Organ Transplantation+"))
S 1	TI((cardi* arrest or cardi* death or circulatory arrest or circulatory death or cardiopulmonary death or (cess* n2 cardiopulmonary) or dead on arrival or maastricht or OHCA or (uncontrolled not “uncontrolled trial*”)) n5 (donat* or donor* or harvest* or "organ transplant*" or procur* or recei* or recover* or rescu* or retriev* or "tissue procurement" or transplant*)) or AB (((cardi* arrest or cardi* death or circulatory arrest or circulatory death or cardiopulmonary death or (cess* n2 cardiopulmonary) or dead on arrival or maastricht or OHCA or (uncontrolled not “uncontrolled trial*”)) n5 (donat* or donor* or harvest* or "organ transplant*" or procur* or recei* or recover* or rescu* or retriev* or "tissue procurement" or transplant*)))

Web of Science Core Collection

Updated by limiting line 10 to years 2022-2023
Science Citation Index Expanded (SCI-EXPANDED)--1900-present
Social Sciences Citation Index (SSCI)--1956-present
Arts & Humanities Citation Index (AHCI)--1975-present
Conference Proceedings Citation Index Science -(CPCI-S)--1990-present
Conference Proceedings Citation Index Social Science & Humanities - (CPCI-SSH)--1990-present
Book Citation Index – Science (BKCI-S)--2008-present
Book Citation Index – Social Sciences & Humanities (BKCI-SSH)--2008-present
Emerging Sources Citation Index (ESCI)--2017-present
Current Chemical Reactions (CCR-EXPANDED)--1985-present

Index Chemicus (IC)--1993-present

10	#8 not #9
9	TI=("animal" or "rat" or "rats" or "mice" or "mouse" or "pig" or "pigs") or AB=("animal" or "rat" or "rats" or "mice" or "mouse" or "pig" or "pigs")
8	#5 and #6 and #7
7	TI=(“act” or "best interest*" or “beneficiar*” or "information shar*" or “challeng*” or ”barrier*” or “facilitate”* or “practical*” or “code*” or “constitution*” or “difficult*” or “ethic*” or “fair*” or “ideal*” or” illegal*” or “immoral*” or “issue*” or “law*” or “legal*” or “legislat*” or “jurisprudence” or “litigat*” or “moral*” or “principl*” or “philosoph*” or “precedent” or “problem*” or (“public” NEAR/3 (“opinion*” or “perception*” or “perceiv*” or “attitude*”)) or “regulat*” or “right*” or “standard*” or “statut*” or “unconscion*” or “unconstitutional” or “unethic*” or “unfair*” or “unlawful” or “value*” or “virtue*” or “virtuous”) OR AB=(“act” or "best interest*" or “beneficiar*” or "information shar*" or “challeng*” or ”barrier*” or “facilitate”* or “practical*” or “code*” or “constitution*” or “difficult*” or “ethic*” or “fair*” or “ideal*” or” illegal*” or “immoral*” or “issue*” or “law*” or “legal*” or “legislat*” or “jurisprudence” or “litigat*” or “moral*” or “principl*” or “philosoph*” or “precedent” or “problem*” or (“public” NEAR/3 (“opinion*” or “perception*” or “perceiv*” or “attitude*”)) or “regulat*” or “right*” or “standard*” or “statut*” or “unconscion*” or “unconstitutional” or “unethic*” or “unfair*” or “unlawful” or “value*” or “virtue*” or “virtuous”)
6	TI=("organ*" or "kidney*" or "liver*" or "heart*" or "lung*" or "multivisceral" or "graft" or "bloc" or “pancreas” or “small intestine” or “bowel”) OR AB=("organ*" or "kidney*" or "liver*" or "heart*" or "lung*" or "multivisceral" or "graft" or "bloc" or “pancreas” or “small intestine” or “bowel”)
5	#1 or #2 or #3
3	TI=(“UDCD*” or “DCD*”) OR AB=(“UDCD*” or “DCD*”)
2	AB=((("cardi* arrest" or "cardi* death" or "circulatory arrest" or "circulatory death" or "dead on arrival" or “Maastricht” or “OHCA” or “uncontrolled” or “cardiopulmonary death” or (cess* near/2 cardiopulmonary))) near/5 ((“donat*” or “donor*” or “harvest*” or “organ transplant*” or “procur*” or “recei*” or “recover”* or “rescu*” or “retriev*” or “tissue procurement” or “transplant*”)))
1	TI=((("cardi* arrest" or "cardi* death" or "circulatory arrest" or "circulatory death" or "dead on arrival" or “Maastricht” or “OHCA” or “uncontrolled” or “cardiopulmonary death” or (cess* near/2 cardiopulmonary))) near/5 ((“donat*” or “donor*” or “harvest*” or “organ transplant*” or “procur*” or “recei*” or “recover”* or “rescu*” or “retriev*” or “tissue procurement” or “transplant*”)))

Scopus

((TITLE-ABS ("cardi* arrest" OR "cardi* death" OR "circulatory arrest" OR "circulatory death" OR "dead on arrival" OR maastricht OR ohca OR uncontrolled OR "cardiopulmonary death" OR (cess* W/2 cardiopulmonary)) W/5 ((donat* OR donor* OR harvest* OR "organ transplant*" OR procur* OR recei* OR recover* OR rescu* OR retriev* OR "tissue procurement" OR transplant*))) OR TITLE-ABS (udcd* OR dcd*)) AND TITLE-ABS (("organ*" OR "kidney*" OR "liver*" OR "heart*" OR "lung*" OR "multivisceral" OR "graft" OR "bloc" OR "pancreas" OR "small intestine" OR "bowel")) AND (TITLE-ABS ((act OR "best

interest*" OR beneficiar* OR "information shar*" OR challeng* OR barrier* OR facilitat* OR practical* OR code* OR constit ution* OR difficult* OR ethic* OR fair* OR ideal* OR illegal* OR immoral* OR issue* OR law* OR legal* OR legislat* OR jurisprudence OR litigat* OR moral* OR principl* OR philosoph* OR precedent OR problem* OR (public W/3 (opinion * OR perception* OR perceiv* OR attitude*)) OR regulat* OR right* OR standar d* OR statut* OR unconscion* OR unconstitutional OR unethic* OR unfair* OR u nlawful OR value* OR virtu*))) AND NOT TITLE-ABS ("animal" OR "rat" OR "rats" OR "mice" OR "mouse" OR "pig" OR "pigs")

Lexis

UDCD
Uncontrolled AND donation AND after AND circulatory AND death
DCD
Donation AND after AND circulatory AND death
Uncontrolled AND organ AND don!
Uncontrolled AND don! AND ethic!
Organ AND Maastricht AND donat!
("cardiac arrest" OR "cardiac death" OR "circulatory arrest" OR "circulatory death") AND don!
("heart arrest" OR "dead on arrival" OR "out of hospital cardiac arrest" OR OHCA) AND don!
(Uncontrolled OR cardiac arrest OR cardiac death OR circulatory arrest OR circulatory death OR OHCA OR "out of hospital cardiac arrest") & (donat! OR donor!) & ("best interest" OR beneficiar! OR "information sharing" OR "public opinion" OR perception! OR attitude!)
(Uncontrolled OR cardiac arrest OR cardiac death OR circulatory arrest OR circulatory death OR OHCA OR "out of hospital cardiac arrest") & (donat! OR donor!) & (challeng! OR barrier! OR facilitat! OR practical! OR difficult! OR problem!)
(Uncontrolled OR cardiac arrest OR cardiac death OR circulatory arrest OR circulatory death OR OHCA OR "out of hospital cardiac arrest")& (donat! OR donor!) & (ethic! OR fair! OR ideal! OR illegal! OR immoral! OR jurisprudence! OR law! OR legal! OR legislat! OR litigat! OR moral! OR philosoph! OR principl! OR regulat! OR unconscion! OR unethic! OR unfair! OR unlaw! OR value! OR virtu!)
(Uncontrolled OR cardiac arrest OR cardiac death OR circulatory arrest OR circulatory death OR OHCA OR "out of hospital cardiac arrest") & (Procur! OR rescu! OR retriev!) & ("best interest" OR beneficiar! OR "information sharing" OR "public opinion" OR perception! OR attitude!)
(Uncontrolled OR cardiac arrest OR cardiac death OR circulatory arrest OR circulatory death OR OHCA OR "out of hospital cardiac arrest") & (Procur! OR rescu! OR retriev!) & (Challeng! OR barrier! OR facilitat! OR practical! OR difficult! OR problem!)
(Uncontrolled OR cardiac arrest OR cardiac death OR circulatory arrest OR circulatory death OR OHCA OR "out of hospital cardiac arrest") & (Procur! OR rescu! OR retriev!) & (Ethic! OR fair! OR ideal! OR illegal! OR immoral! OR jurisprudence! OR law! OR legal! OR legislat! OR litigat! OR moral! OR philosoph! OR principl! OR regulat! OR unconscion! OR unethic! OR unfair! OR unlaw! OR value! OR virtu!)

(Uncontrolled OR cardiac arrest OR cardiac death OR circulatory arrest OR circulatory death OR OHCA OR “out of hospital cardiac arrest”) & (Transplant! OR harvest!) & (“best interest” OR beneficiar! OR “information sharing” OR “public opinion” OR perception! OR attitude!)
(uncontrolled OR cardiac arrest OR cardiac death OR circulatory arrest OR circulatory death OR OHCA OR “out of hospital cardiac arrest”) & (Transplant! OR harvest!) & (challeng! OR barrier! OR facilitat! OR practical! OR difficult! OR problem!)
(uncontrolled OR cardiac arrest OR cardiac death OR circulatory arrest OR circulatory death OR OHCA OR “out of hospital cardiac arrest”) & (Transplant! OR harvest!) & (ethic! OR fair! OR ideal! OR illegal! OR immoral! OR jurisprudence! OR law! OR legal! OR legislat! OR litigat! OR moral! OR philosoph! OR principl! OR regulat! OR unconscion! OR unethic! OR unfair! OR unlaw! OR value! OR virtu!)

WestLaw

UDCD
Uncontrolled donation after circulatory death
DCD
Donation after circulatory death
Uncontrolled & organ & don!
Uncontrolled & don! & ethic!
Organ & Maastricht & donat!
"cardi! arrest" OR "cardi! death" OR "circulat! arrest" OR "circulat! death" & don!
“heart arrest” OR “dead on arrival” OR “out of hospital cardiac arrest” OR OHCA & don!
Uncontrolled & donat! OR donor! & “best interest” OR beneficiar! OR “information sharing” OR “public opinion” OR perception! OR attitude!
Uncontrolled & donat! OR donor! & challeng! OR barrier! OR facilitat! OR practical! OR difficult! OR problem!
Uncontrolled & donat! OR donor! & ethic! OR fair! OR ideal! OR illegal! OR immoral! OR jurisprudence! OR law! OR legal! OR legislat! OR litigat! OR moral! OR philosoph! OR principl! OR regulat! OR unconscion! OR unethic! OR unfair! OR unlaw! OR value! OR virtu!
Uncontrolled & Procur! OR rescu! OR retriev! & “best interest” OR beneficiar! OR “information sharing” OR “public opinion” OR perception! OR attitude!
Uncontrolled & Procur! OR rescu! OR retriev! & Challeng! OR barrier! OR facilitat! OR practical! OR difficult! OR problem!
Uncontrolled & Procur! OR rescu! OR retriev! & Ethic! OR fair! OR ideal! OR illegal! OR immoral! OR jurisprudence! OR law! OR legal! OR legislat! OR litigat! OR moral! OR philosoph! OR principl! OR regulat! OR unconscion! OR unethic! OR unfair! OR unlaw! OR value! OR virtu!
Uncontrolled & Transplant! OR harvest! & “best interest” OR beneficiar! OR “information sharing” OR “public opinion” OR perception! OR attitude!
Uncontrolled & Transplant! OR harvest! & challeng! OR barrier! OR facilitat! OR practical! OR difficult! OR problem!
Uncontrolled & Transplant! OR harvest! & Ethic! OR fair! OR ideal! OR illegal! OR immoral! OR jurisprudence! OR law! OR legal! OR legislat! OR litigat! OR moral! OR philosoph! OR principl! OR regulat! OR unconscion! OR unethic! OR unfair! OR unlaw! OR value! OR virtu!

“cardiac arrest" OR "cardiac death" OR "circulatory arrest" OR "circulatory death" & donat! OR donor! & “best interest” OR beneficiar! OR “information sharing” OR “public opinion” OR perception! OR attitude!
“cardiac arrest" OR "cardiac death" OR "circulatory arrest" OR "circulatory death" & donat! OR donor! & challeng! OR barrier! OR facilitat! OR practical! OR difficult! OR problem!
“cardiac arrest" OR "cardiac death" OR "circulatory arrest" OR "circulatory death" & donat! OR donor! & ethic! OR fair! OR ideal! OR illegal! OR immoral! OR jurisprudence! OR law! OR legal! OR legislat! OR litigat! OR moral! OR philosoph! OR principl! OR regulat! OR unconscion! OR unethic! OR unfair! OR unlaw! OR value! OR virtu!
“cardiac arrest" OR "cardiac death" OR "circulatory arrest" OR "circulatory death" & procur! OR rescu! OR retriev! & “best interest” OR beneficiar! OR “information sharing” OR “public opinion” OR perception! OR attitude!
“cardiac arrest" OR "cardiac death" OR "circulatory arrest" OR "circulatory death" & procur! OR rescu! OR retriev! & challeng! OR barrier! OR facilitat! OR practical! OR difficult! OR problem!
“cardiac arrest" OR "cardiac death" OR "circulatory arrest" OR "circulatory death" & procur! OR rescu! OR retriev! & ethic! OR fair! OR ideal! OR illegal! OR immoral! OR jurisprudence! OR law! OR legal! OR legislat! OR litigat! OR moral! OR philosoph! OR principl! OR regulat! OR unconscion! OR unethic! OR unfair! OR unlaw! OR value! OR virtu!
“cardiac arrest" OR "cardiac death" OR "circulatory arrest" OR "circulatory death" & transplant! OR harvest! & “best interest” OR beneficiar! OR “information sharing” OR “public opinion” OR perception! OR attitude!
“cardiac arrest" OR "cardiac death" OR "circulatory arrest" OR "circulatory death" & transplant! OR harvest! & challeng! OR barrier! OR facilitat! OR practical! OR difficult! OR problem!
“cardiac arrest" OR "cardiac death" OR "circulatory arrest" OR "circulatory death" & transplant! OR harvest! & ethic! OR fair! OR ideal! OR illegal! OR immoral! OR jurisprudence! OR law! OR legal! OR legislat! OR litigat! OR moral! OR philosoph! OR principl! OR regulat! OR unconscion! OR unethic! OR unfair! OR unlaw! OR value! OR virtu!
“heart arrest” OR “dead on arrival” OR “out of hospital cardiac arrest” OR OHCA & donat! OR donor! & “best interest” OR beneficiar! OR “information sharing” OR “public opinion” OR perception! OR attitude!
“heart arrest” OR “dead on arrival” OR “out of hospital cardiac arrest” OR OHCA & donat! OR donor! & challeng! OR barrier! OR facilitat! OR practical! OR difficult! OR problem!
“heart arrest” OR “dead on arrival” OR “out of hospital cardiac arrest” OR OHCA & donat! OR donor! & Ethic! OR fair! OR ideal! OR illegal! OR immoral! OR jurisprudence! OR law! OR legal! OR legislat! OR litigat! OR moral! OR philosoph! OR principl! OR regulat! OR unconscion! OR unethic! OR unfair! OR unlaw! OR value! OR virtu!
“heart arrest” OR “dead on arrival” OR “out of hospital cardiac arrest” OR OHCA & procur! OR rescu! OR retriev! & “best interest” OR beneficiar! OR “information sharing” OR “public opinion” OR perception! OR attitude!
“heart arrest” OR “dead on arrival” OR “out of hospital cardiac arrest” OR OHCA & procur! OR rescu! OR retriev! & challeng! OR barrier! OR facilitat! OR practical! OR difficult! OR problem!
“heart arrest” OR “dead on arrival” OR “out of hospital cardiac arrest” OR OHCA & procur! OR rescu! OR retriev! & ethic! OR fair! OR ideal! OR illegal! OR immoral! OR jurisprudence! OR law! OR legal! OR legislat! OR litigat! OR moral! OR philosoph! OR

principl! OR regulat! OR unconscion! OR unethic! OR unfair! OR unlaw! OR value! OR virtu!
“heart arrest” OR “dead on arrival” OR “out of hospital cardiac arrest” OR OHCA & transplant! OR harvest! & “best interest” OR beneficiar! OR “information sharing” OR “public opinion” OR perception! OR attitude!
“heart arrest” OR “dead on arrival” OR “out of hospital cardiac arrest” OR OHCA & transplant! OR harvest! & challeng! OR barrier! OR facilitat! OR practical! OR difficult! OR problem!
“heart arrest” OR “dead on arrival” OR “out of hospital cardiac arrest” OR OHCA & transplant! OR harvest! & Ethic! OR fair! OR ideal! OR illegal! OR immoral! OR jurisprudence! OR law! OR legal! OR legislat! OR litigat! OR moral! OR philosoph! OR principl! OR regulat! OR unconscion! OR unethic! OR unfair! OR unlaw! OR value! OR virtu!
